# Identifying factors influencing contraceptive use in Bangladesh: evidence from BDHS 2014 data

**DOI:** 10.1186/s12889-018-5098-1

**Published:** 2018-01-30

**Authors:** MB Hossain, MHR Khan, F Ababneh, JEH Shaw

**Affiliations:** 1 BRAC Research and Evaluation Division, BRAC Center, 75 Mohakhali, Dhaka, 1212 Bangladesh; 2 Institute of Statistical Research and Training (ISRT), University of Dhaka, Dhaka, 1000 Bangladesh; 3 Department of Mathematics and Statistics, Sultan Qaboos University, Muscat, Oman; 4 Department of Mathematics, Al-Hussein Bin Talal University, Maan, Jordan; 5 Department of Statistics, University of Warwick, Coventry, UK

**Keywords:** Bangladesh Demographic and Health Survey (BDHS), Sampling weight, Divisions, Mixed effect, Generalized estimating equation (GEE)

## Abstract

**Background:**

Birth control is the conscious control of the birth rate by methods which temporarily prevent conception by interfering with the normal process of ovulation, fertilization, and implantation. High contraceptive prevalence rate is always expected for controlling births for those countries that are experiencing high population growth rate. The factors that influence contraceptive prevalence are also important to know for policy implication purposes in Bangladesh. This study aims to explore the socio-economic, demographic and others key factors that influence the use of contraception in Bangladesh.

**Methods:**

The contraception data are extracted from the 2014 Bangladesh Demographic and Health Survey (BDHS) data which were collected by using a two stage stratified random sampling technique that is a source of nested variability. The nested sources of variability must be incorporated in the model using random effects in order to model the actual parameter effects on contraceptive prevalence. A mixed effect logistic regression model has been implemented for the binary contraceptive data, where parameters are estimated through generalized estimating equation by assuming exchangeable correlation structure to explore and identify the factors that truly affect the use of contraception in Bangladesh.

**Results:**

The prevalence of contraception use by currently married 15–49 years aged women or their husbands is 62.4%. Our study finds that administrative division, place of residence, religion, number of household members, woman’s age, occupation, body mass index, breastfeeding practice, husband’s education, wish for children, living status with wife, sexual activity in past year, women amenorrheic status, abstaining status, number of children born in last five years and total children ever died were significantly associated with contraception use in Bangladesh.

**Conclusions:**

The odds of women experiencing the outcome of interest are not independent due to the nested structure of the data. As a result, a mixed effect model is implemented for the binary variable ‘contraceptive use’ to produce true estimates for the significant determinants of contraceptive use in Bangladesh. Knowing such true estimates is important for attaining future goals including increasing contraception use from 62 to 75% by 2020 by the Bangladesh government’s Health, Population & Nutrition Sector Development Program (HPNSDP).

**Electronic supplementary material:**

The online version of this article (10.1186/s12889-018-5098-1) contains supplementary material, which is available to authorized users.

## Background

The original meaning of the word *contraception* is birth control technique [1]. Contraception is defined generally as the intentional prevention of conception or impregnation during sexual activity, through man made means such as the use of various devices, agents, drugs, sexual practices or surgical procedures. The main goal of these methods is to prevent the sperm from reaching the ovum by using condoms, diaphragms etc., inhibiting ovulation, preventing implantation and so on. It was known that, in Bangladesh, almost half of all deaths in women of child-bearing age were caused by problems of pregnancy and childbirth and unsafe abortion [2, 3]. Moreover, women under the age of 18 are more likely to die in childbirth because their bodies are not fully grown, they are not physically or emotionally (even if financially) ready to carry and care for a child, and their babies tend to have low birth weight and face a variety of illnesses which cause a greater chance of dying before reaching their second birthday [4, 5]. Many scientists found that most of these complications, especially pregnancy complications and deaths, could be prevented by proper family planning [4] because family planning has great advantages for the mother, children, father and the family. Contraception is one of the key components of family planning, and can therefore help stave off poor health of women and children, as well as household food insecurity. Use of contraception such as condoms can also help protect against the spread of sexually transmitted infections including HIV. Moreover, hormonal methods can help with irregular bleeding and pain during a woman’s monthly bleeding. It is also evident that to avoid unwanted pregnancies, to choose the size of family and improve the spacing between the births of children, to reduce abortion and to allow spontaneous sex, contraceptives are inevitable and convenient for both men and women [6].

**Fig. 1:**
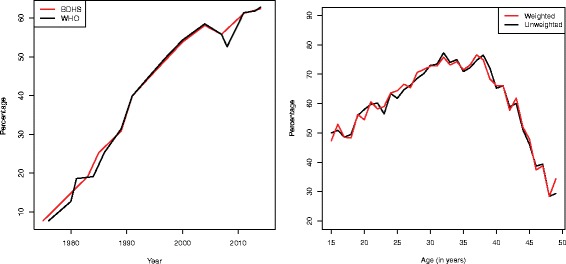
Prevalence of contraception use by currently married 15–49 years aged women in Bangladesh (left panel) and age-specific prevalence of contraception use by currently married 15–49 years women in Bangladesh (right panel) (Note that before 2007 survey, the contraceptive use data were collected from 10–49 years of women)

Many United Nations member countries, particularly those in the developed world, have strong family planning programs and nine out of every ten contraceptive users in the world rely on modern methods of contraception [7]. For the world as a whole, 64% currently married or in-union women aged 15–49 years currently use some form of contraception, whereas the prevalence is significantly lower in the least developed countries (40%) [7–9]. The current prevalence of contraceptive use in Bangladesh (62%) is very close to the world contraceptive use prevalence (64%) while the prevalence is higher than in other South Asian countries such as India (58%), Nepal (50%), Pakistan (35%) and Afghanistan (23%) [10–15]. However, a huge jump in the use of contraception among married women in Bangladesh has taken place since 1975 (8% in 1975 to 62% in 2014) [11]. Most recently, the Bangladesh Demographic Health Survey (BDHS) 2014 had shown that contraceptive use increased by 6% from 56% in 2007 to 62% in 2014 while it increased by only 1% in the past three years (from 61% in 2011 to 62% in 2014) [10, 11]. The World Health Organization (WHO) had also shown a roughly similar pattern of contraception use for currently married 15–49 year old women of the region (Fig. 1).

Since the growing use of contraceptive methods is associated not only with improvement of health related outcomes such as reduced maternal and child mortality, but also with improvements in schooling and economic outcomes especially for girls and women [7], various global and local organizations, private sectors and governments still aiming to maximise contraceptive use. To achieve this, as an outcome of the 2012 London Summit on Family Planning, 69 of the world’s poorest countries set an agenda called Family Planning 2020 (FP2020), aiming to increase access to family planning information, services and supplies to 120 million more women and girls in 69 of the world’s poorest countries by 2020 [16]. For Bangladesh, one of the main aims was set to increase contraceptive prevalence from 62 to 75% by 2020 [16]. The Health, Population and Nutrition Sector Development Program (HPNSDP) of Bangladesh also set strategic plans to make family planning services available, accessible, acceptable and affordable to all men and women (of reproductive age) in order to obtain an overall use of family planning of 80% by 2021 [11, 16–18]. This is why many researchers and policy makers had shown interest in exactly which factors affect contraceptive prevalence in Bangladesh.

Many past studies including [19–21] found that the use of contraception has increased in all regions in Bangladesh since 1975 and there remains still substantial geographical variation in contraceptive use within the region. Khan [22] had shown that if women are adequately counselled and supported to sustain their use of more effective contraceptive methods, they will need less medical attention, have fewer unintended pregnancies and reduce the workload for medical clinics. However, since multilevel models are generally capable of identifying hierarchical variation among regions, many authors [21, 23–27] also considered hierarchical variation and multilevel analysis to identify the significant variation in contraceptive use at administrative division or district level and significant socio-demographic factors that changed the pattern of contraceptive use. Meanwhile, using binary contraceptive prevalence data from BDHS 2004 data, Khan and Shaw [21, 25] had shown that the multilevel model performs well and avoids either underestimation or overestimation when the structure of data is naturally hierarchical. Also, in Bangladesh, the approval of contraceptive methods is largely influenced by the male member of couples, and the use of contraception also largely depends on the knowledge and attitudes of males towards modern contraception like contraceptive pills, implants, injectable, intrauterine device, male and female condoms, vasectomy etc. [28]. Nowadays, men’s contraceptive knowledge and their attitude to modern contraception is high in Bangladesh and many factors such as women’s age, education and socio-economic status, number of living children in the family, children ever died, male’s higher knowledge and positive attitude towards modern contraceptives were significantly associated with contraception use [21, 24, 25, 28–31].

**Fig. 2:**
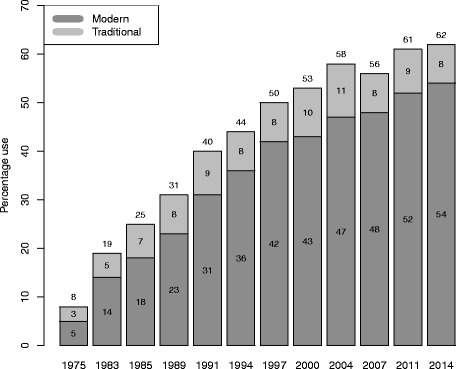
Trends in contraception use among currently married women (Note that before 2007 survey, the contraceptive use data were collected from 10–49 years of women)

BDHS data are naturally hierarchical with data collected using two stage stratified random sampling. To ensure accurate representation of nationwide data, BDHS data require sampling weights where sample weights were not necessary for estimating relationships such as regression and correlation coefficients [32]. This is why after adjusting sampling weights and hierarchy structure of the data, still many researchers and policy makers will want to know exactly the factors that truly affect contraceptive use in Bangladesh. But in the context of mixed effect model, statistical analysis of contraception had rarely been done for Bangladesh. However, although the use of contraception increased considerably in this region, from 8% in 1975 to 62% in 2014; in the last decade it increased by only 4% (Fig. 2). This presents a challenging situation for policy makers to reach the HPNSDP and FP2020 goal of a 13% increase in contraception use in the next few years. Given the context of high fertility in this region, to meet these goals and show a path to the policy makers, our study seeks to explore the socio-economic, demographic and other woman- and/or husband-related factors that truly affect the use of contraception by currently married 15–49 years aged women in Bangladesh.

## Methods

### Study site and participants

A secondary dataset from the Bangladesh Demographic Health Survey (BDHS) 2014 was used for this study Additional file 1. The sample for the 2014 BDHS was a nationally representative cross sectional survey conducted between June and November 2014, covering the entire population of Bangladesh. The survey was based on a two-stage stratified sample of households. In the first stage, 600 enumerations areas (EAs) were selected with probability proportional to EA size, with 207 EAs from urban areas and 393 from rural areas, where an EA is either a village, a group of small villages, or a part of a large village [11]. In the second stage, a systematic sample of 30 households on average was selected per EA to provide statistically reliable estimates of the key demographic and health variables for the country as a whole, for urban and rural areas separately and each of the seven divisions which are the administrative regions of Bangladesh [11]. The 2014 BDHS used three types of questionnaires: a household questionnaire, a women questionnaire and a community questionnaire. For the women questionnaire, a total of 18,000 ever married and 16858 currently married (when conducting the interview) women aged between 15–49 years were interviewed. For our study, we considered only currently married women to ensure similar prevalence as BDHS 2014 reported and to compare the findings with the findings from others studies.

### Variables assessed and measured

Women were asked questions on the following topics: Background characteristics such as age, level of education, religion, media exposure on family planning; Reproductive history; Use and source(s) of family planning methods; Antenatal, delivery, postnatal and newborn care; Breastfeeding and infant feeding practices; Child immunizations and illnesses; Marriage; Fertility preferences; Husband’s background and respondent’s work; Awareness of AIDS and other sexually transmitted infections [11].

### Outcome measurements

**Fig. 3:**
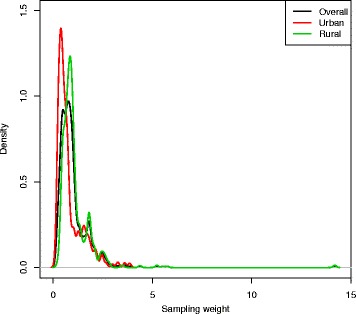
Distribution of individual sampling weight

The use of contraceptive or contraceptive prevalence is the percentage of 15–49 years aged women who are currently using, or whose sexual partner is currently using, at least one method of contraception, regardless of the method used [33]. In BDHS surveys, current use of contraception is defined as the proportion of currently married women who report that they are using a family planning method at the time of the survey [11, 32]. Since any analysis using the 2014 BDHS data requires that sampling weights be applied to ensure the applicability of the survey results at the national and domain levels, we considered the weight variable while constructing the outcome variable. The distribution of individual sampling weight is shown in Fig. 3. The brief calculation and explanation of weight variable can be found in BDHS 2014 report [11].

### Statistical analysis

Descriptive statistics methods were applied to assess the distribution of the full range of variables using appropriate cut-off, dichotomous or categorical variables. Chi-square tests were used to relate the prevalence of contraceptive use to the characteristics of households, women, and/or their husbands. Since the data are nested in nature, it is reasonable to consider the nested sources of variability by considering random effects along with the usual fixed effects to identify the factors that truly affect the use of contraception. However, each of the variables was assessed individually with the outcome variable through simple (also called single-covariate) mixed effect logistic regression modelling [34, 35]. In that case, EAs were considered as random effect with exchangeable correlation structure where the parameters of the model were estimated through the generalized estimating equation (GEE) approach defined by Liang and Zeger [36]. Finally, each of the variables was fed into the mixed effect multiple logistic regression model to adjust for potential confounding, modelling EA as a random effect and assuming an exchangeable correlation structure.

The odds ratio (OR) and corresponding 95% confidence intervals (95% CIs) were estimated with statistical significance defined as *α*≤0.05 and the final model was selected by the strategy followed by Agresti [37]. All analyses were performed using Stata (Version 12.0) and R (Version 3.3.1, RStudio version: 1.0.136).

## Results

### Prevalence of contraceptive use

**Fig. 4:**
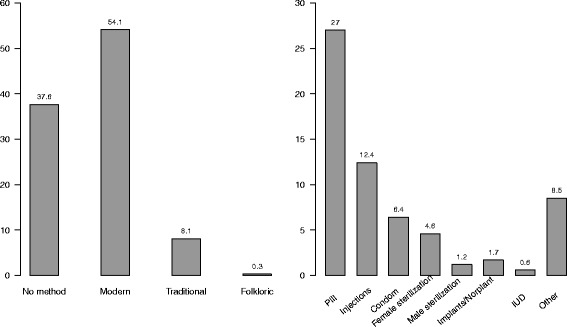
Choice of current contraceptive method type by currently married 15–49 years women (left panel) and Methods of contraception use by currently married 15–49 years aged women or their respective husband (right panel) in Bangladesh

The prevalence of contraceptive use by the household background characteristics of the study population is presented in Table 1. It was found that the weighted prevalence of contraception use by currently married 15–49 years women was 62.4% among the total of 16858 women, where about 54% women were using modern methods and 8% were using traditional methods (Fig. 2 and left panel of Fig. 4). The pill is by far the most used method (27%), followed by injectables (12.4%), condoms (6.4%) and male or female sterilization (5.8%) (right panel of Fig. 4). It is noted that use of contraception varied across administration divisions. The prevalence is highest for Rangpur and Rajshahi divisions (69.8% and 69.4% respectively) while it was very low for Sylhet and Chittagong divisions (47.8% and 55.0% respectively). There was found to be a higher prevalence of contraception use in urban areas (65.9%) compared to rural areas (61.1%). Non-Muslims women were using more contraception than Muslims (69.4% versus 61.7%). Women having a male household head were using more contraception than those with a female household head (65.3% and 32.3% respectively). It is also noticed that women from households with 4 or more members had higher prevalence of contraception use than women from households with fewer than 4 members, while there was no difference in contraception use levels between different wealth quintiles.

**Table 1:** Background characteristics of the households

Variable	Category	Total	Contraception use		*p*-value
		n	No, n (%)	Yes, n (%)	
Total		16858 (100%)	6331 (37.6)	10527 (62.4)	
Division	Dhaka	5857	2169 (37.0)	3687 (63.0)	0.001
	Chittagong	3121	1405 (45.0)	1717 (55.0)	
	Rajshahi	2007	615 (30.6)	1392 (69.4)	
	Khulna	1729	569(32.9)	1160 (67.1)	
	Barisal	1051	386 (36.7)	665 (63.3)	
	Rangpur	1946	588 (30.2)	1358 (69.8)	
	Sylhet	1147	599 (52.2)	548 (47.8)	
Residence	Urban	4709	1607 (34.1)	3102 (65.9)	0.001
	Rural	12149	4723 (38.9)	7425 (61.1)	
Religion	Non-Muslim	100	511 (30.6)	1160 (69.4)	0.001
	Muslim	16758	5820 (38.3)	9367 (61.7)	
Sex of household head	Female	1458	988 (67.7)	471 (32.3)	0.001
	Male	15400	5343 (34.7)	10057 (65.3)	
Number of household member	< 4	3188	1527 (47.9)	1661 (52.1)	0.001
	≥4	13670	4803 (35.1)	8866 (64.9)	
Wealth quintile	Poorest	3097	1159 (37.4)	1938 (62.6)	0.783
	Poorer	3223	1187 (36.8)	2036 (63.2)	
	Middle	3394	1267 (37.3)	2127 (62.7)	
	Richer	3556	1384 (38.9)	2172 (61.1)	
	Richest	3587	1333 (37.2)	2254 (62.8)	

*p*-value ≤0.001 are treated as 0.001

Current contraception use varies considerably by age (Table 2, right panel of Fig. 1). The prevalence of contraception use was highest for women aged 30–34 years (73.7%), followed by those aged 35–39 (72.9%). The 15–19 years women had the second lowest contraceptive use (51.2%), while the oldest group (45–49 years) had the lowest use (38.0%). The oldest group were more likely to use periodic abstinence whereas women of other age groups mostly used the pill. Although the prevalence of contraception use did not vary by women’s education, their occupation played a vital role. Employed women used more contraception (68.1%) compare to unemployed (59.8%). It was also found that underweight and obese women used less contraception (59.5% and 58.4% respectively) than normal weight women (63.4%).

**Table 2:** Background characteristics of the targeted women

Variable	Category	Total	Contraception use		*p*-value
		n	No, n (%)	Yes, n (%)	
Total		16858 (100%)	6331 (37.6)	10527 (62.4)	
Age (in years)	15-19	1984	969 (48.8)	1015 (51.2)	0.001
	20-24	3166	1292 (40.8)	1874 (59.2)	
	25-29	3249	1050 (32.3)	2200 (67.7)	
	30-34	2919	767 (26.3)	2152 (73.7)	
	35-39	2153	583 (27.1)	1570 (72.9)	
	40-44	1874	733 (39.1)	1140 (60.9)	
	45-49	1512	937 (62.0)	575 (38.0)	
Education	No education	3949	1519 (38.5)	2429 (61.5)	0.289
	Primary	4916	1812 (36.9)	3104 (63.1)	
	Secondary	6503	2474 (38.0)	4029 (62.0)	
	Higher	1490	525 (35.2)	965 (64.8)	
Occupation	Unemployed	11487	4617 (40.2)	6870 (59.8)	0.001
	Employed	5371	1714 (31.9)	3657 (68.1)	
BMI	< 18.5	2953	1196 (40.5)	1756 (59.5)	0.005
	18.5-24.9	9774	3577 (36.6)	6197 (63.4)	
	25-29.9	3262	1196 (36.7)	2067 (63.3)	
	≥ 30	869	362 (41.6)	507 (58.4)	
Currently breastfeeding	No	13071	5135 (39.3)	7936 (60.7)	0.001
	Yes	3787	1195 (31.6)	2591 (68.4)	

*p*-value ≤0.001 are treated as 0.001

The background characteristics of others factors related to couples and associated with contraception use is described in Table 3. Women who were older than, or no more than 5 years younger than, their husband had slightly higher prevalence of contraception use (63.8%) than 6–10 years and 10 years more aged husband (63.4% and 60.0% respectively). Women whose husband had higher education showed slightly higher contraceptive use (65.1%) compared to those with an uneducated husband (62.7%). Also, women whose husband was employed had higher prevalence of contraception use (62.5%) than those with an unemployed husband (56.8%). It was found that when the husband currently lives with her, the use of contraception (67.9%) was significantly higher than when the husband was staying elsewhere (24.5%). Interestingly, it was found that when the husband wanted fewer children, the use of contraception was higher (65.9%) compared to when both wanted the same number (60.7%) or when the husband wanted more (58.3%). Women who currently do not breastfeed, are amenorrheic, abstaining, or sexually less active in the past year had significantly lower prevalence of contraception use (60.7%, 24.1%, 13.2% and 22.5% respectively). It was also found that there was no significant relationship between contraception use and exposure to media messages on family planning.

**Table 3:** Background characteristics of other factors of targeted women and/or her husband

Variable	Category	Total	Contraception use		*p*-value
		n	No, n (%)	Yes, n (%)	
Total		16858 (100%)	6331 (37.6)	10527 (62.4)	
Husband age difference with respondent	Woman older or same age	261	96 (36.6)	166 (63.4)	0.012
	1-5 years older	4543	1641 (36.1)	2901 (63.9)	
	6-10 years older	6705	2456 (36.6)	4249 (63.4)	
	> 10 years older	5349	2138 (40.0)	3211 (60.0)	
Husband education level	No education	4715	1757 (37.3)	2958 (62.7)	0.013
	Primary	4680	1724 (36.8)	2956 (63.2)	
	Secondary	5085	2019 (39.7)	3066 (60.3)	
	Higher	2379	831 (34.9)	1548 (65.1)	
Husband occupation	Unemployed	116	50 (43.2)	66 (56.8)	0.179
	Employed	16742	6281 (37.5)	10461 (62.5)	
Currently residing with husband	Living with her	14755	4742 (32.1)	10013 (67.9)	0.001
	Staying elsewhere	2102	1588 (75.5)	514 (24.5)	
Desire for child	Both desire same number	12514	4918 (39.3)	7596 (60.7)	0.001
	Husband desire more	1776	741 (41.7)	1035 (58.3)	
	Husband desire fewer	1122	383 (34.1)	739 (65.9)	
	Don’t know	452	287 (63.5)	165 (36.5)	
	Missing	994	2 (0.2)	992 (99.8)	
Currently amenorrheic	No	16055	5721 (35.6)	10334 (64.4)	0.001
	Yes	803	609 (75.9)	194 (24.1)	
Currently abstaining	No	16366	5903 (36.1)	10463 (63.9)	0.001
	Yes	492	427 (86.8)	65 (13.2)	
Sexually active in past year	No or moderate	2924	2265 (77.5)	659 (22.5)	0.001
	Yes or highly	13934	4066 (29.2)	9868 (70.8)	
Total children ever born	0-1	5358	2720 (50.8)	2637 (49.2)	0.001
	2-3	7712	2144 (27.8)	5568 (72.2)	
	4+	3788	1466 (38.7)	2322 (61.3)	
Number of living children	0	1707	1254 (73.5)	453 (26.5)	0.001
	1	3991	1631 (40.9)	2361 (59.1)	
	2	4956	1358 (27.4)	3598 (72.6)	
	3+	6203	2088 (33.7)	4115 (66.3)	
Number of child born in last five years	0	9964	4236 (42.5)	5728 (57.5)	0.001
	1	5874	1711 (29.1)	4163 (70.9)	
	2+	1020	384 (37.6)	636 (62.4)	
Total children ever died	0	14,067	5190 (36.9)	8876 (63.1)	0.001
	1	2,163	837 (38.7)	1326 (61.3)	
	2+	628	303 (48.2)	325 (51.8)	
Exposure to media message on FP	No exposure	13426	5088 (37.9)	8338 (62.1)	0.163
	At least some exposure	3432	1243 (36.2)	2189 (63.8)	

*p*-value ≤0.001 are treated as 0.001

### Factors associated with contraceptive use

Individual assessment of each of the covariates with respect to use of contraception are presented in Table ??. Significant variation was seen according to both administrative division and geographical location (residence). In addition, religion, sex of household head, number of household members, household wealth quintile, woman’s age, education, occupation, body mass index (BMI), husband’s level of education, whether or not husband was currently living with wife, sexual activity in past year, desire for children, breastfeeding practice, amenorrheic status, abstaining status, total child ever born, number of living children, number of children born in last five years, and total number of children ever died, show moderate to high significant association with current contraception use. On the other hand, woman’s education, age difference with husband, and media exposure show no more independently significant association with use of contraception.

**Table 4:** Estimated effects and corresponding 95% CI of mixed effect logistic regression model for the use of contraception variable

Variable	Category	COR	*p*-value	95% CI	AOR	*p*-value	95% CI
Constant					0.15	0.001	0.11-0.20
Division	Dhaka	1.00			1.00		
	Chittagong	0.69	0.001	0.60-0.80	0.83	0.031	0.70-0.98
	Rajshahi	1.33	0.001	1.13-1.56	1.31	0.003	1.10-1.56
	Khulna	1.20	0.027	1.02-1.40	1.29	0.004	1.08-1.54
	Barisal	0.95	0.554	0.81-1.12	1.17	0.091	0.97-1.41
	Rangpur	1.33	0.001	1.13-1.56	1.17	0.087	0.98-1.39
	Sylhet	0.55	0.001	0.47-0.64	0.42	0.001	0.34-0.50
Residence	Urban	1.00			1.00		
	Rural	0.80	0.001	0.72-0.89	0.77	0.001	0.69-0.86
Religion	Non-Muslim	1.00			1.00		
	Muslim	0.83	0.003	0.73-0.94	0.84	0.026	0.73-0.98
Sex of household head	Female	1.00			Not retained in the final model		
	Male	3.38	0.001	3.03-3.78			
Number of household members	<4	1.00			1.00		
	≥ 4	1.95	0.001	1.81-2.11	1.83	0.001	1.66-2.02
Wealth quintile	Poorest	1.00			Not retained in the final model		
	Poorer	0.98	0.653	0.88-1.08			
	Middle	0.89	0.025	0.80-0.98			
	Richer	0.81	0.001	0.73-0.91			
	Richest	0.78	0.001	0.70-0.88			
Age (in years)	15-19	1.00			1.00		
	20-24	1.38	0.001	1.23-1.54	1.27	0.001	1.10-1.47
	25-29	1.82	0.001	1.62-2.03	1.87	0.001	1.62-2.16
	30-34	2.73	0.001	2.42-3.08	3.37	0.001	2.89-3.94
	35-39	2.47	0.001	2.18-2.81	3.23	0.001	2.73-3.81
	40-44	1.38	0.001	1.22-1.57	1.78	0.001	1.50-2.10
	45-49	0.53	0.001	0.46-0.61	0.67	0.001	0.56-0.80
Education	No education	1.00			Not retained in the final model		
	Primary	1.10	0.032	1.01-1.20			
	Secondary	1.03	0.422	0.95-1.13			
	Higher	1.04	0.503	0.92-1.18			
Occupation	Unemployed	1.00			1.00		
	Employed	1.42	0.001	1.32-1.53	1.26	0.001	1.15-1.37
BMI	18.5-24.9	1.00			1.00		
	<18.5	0.89	0.009	0.82-0.97	0.90	0.066	0.81-1.01
	25-29.9	0.97	0.501	0.90-1.05	0.91	0.068	0.82-1.01
	≥30	0.75	0.001	0.65-0.86	0.71	0.001	0.60-0.84
Currently breastfeeding	No	1.00			1.00		
	Yes	1.50	0.001	1.39-1.63	2.47	0.001	2.12-2.87
Husband’s age difference with wife	Woman older/same age	1.00			Not retained in the final model		
	1-5 years older	1.06	0.660	0.82-1.37			
	6-10 years older	1.07	0.622	0.83-1.38			
	> 10 years older	0.94	0.657	0.73-1.22			
Variable	Category	COR	*p*-value	95% CI	AOR	*p*-value	95% CI
Husband’s education level	No education	1.00			1.00		
	Primary	1.04	0.421	0.95-1.13	1.15	0.008	1.04-1.28
	Secondary	0.85	0.001	0.78-0.93	1.12	0.044	1.01-1.25
	Higher	0.95	0.343	0.86-1.05	1.28	0.001	1.12-1.46
Husband’s occupation	Unemployed	1.00			Not retained in the final model		
	Employed	1.23	0.277	0.85-1.78			
Currently residing with husband	Staying elsewhere	1.00			1.00		
	Living with her	5.60	0.001	5.05-6.21	2.78	0.001	2.41-3.21
Sexually active in past year	No or moderate	1.00			1.00		
	Yes or highly	7.43	0.001	6.78-8.15	4.97	0.001	4.38-5.65
Desire for child	Both desire same number	1.00			1.00		
	Husband desires more	1.02	0.693	0.92-1.13	1.03	0.595	0.91-1.17
	Husband desires fewer	1.25	0.001	1.10-1.42	1.23	0.007	1.06-1.44
Currently amenorrheic	No	1.00			1.00		
	Yes	0.17	0.001	0.14-0.20	0.06	0.001	0.04-0.07
Currently abstaining	No	1.00			1.00		
	Yes	0.08	0.001	0.06-0.10	0.16	0.001	0.10-0.23
Total children ever born	0-1	1.00			Not retained in the final model		
	2-3	2.50	0.001	2.33-2.69			
	4+	1.78	0.001	1.64-1.94			
Number of living children	0	1.00			Not retained in the final model		
	1	3.88	0.001	3.44-4.38			
	2	6.56	0.001	5.82-7.40			
	3+	5.58	0.001	4.97-6.27			
Number of child born in last five years	0	1.00			1.00		
	1	1.93	0.001	1.80-2.06	2.44	0.001	2.17-2.73
	2+	1.51	0.001	1.32-1.74	2.12	0.001	1.69-2.64
Total children ever died	0	1.00			1.00		
	1	0.93	0.107	0.85-1.02	0.86	0.014	0.76-0.97
	2+	0.65	0.001	0.56-0.76	0.72	0.002	0.58-0.88
Exposure to media message on FP	No exposure	1.00			Not retained in the final model		
	At least some exposure	1.05	0.250	0.97-1.13			

COR = Crude odds ratio, AOR = Adjusted odds ratio and *p*-value ≤ 0.001 are treated as 0.001

Finally, the adjusted estimated effects for the factors associated with contraception use are presented in Table ??. We note that there were division specific regional effects on contraception use. Furthermore, residence, religion, number of household members, woman’s age, occupation, BMI, husband’s level of education, whether or not husband currently lives with wife, sexual activity in past year, woman’s breastfeeding practice, amenorrheic status, abstaining status, number of children born in the last five years, and total child ever died remain significant factors associated with use of contraception. A woman from Rajshahi or Khulna division had approximately a 30% higher chance of contraception use than a woman from Dhaka division (AOR: 1.31 and 1.29, 95% CI: 1.10-1.56 and 1.08-1.54 respectively). On the other hand, a woman from Chittagong division had 17% less chance and a woman from Sylhet division had less than half the chance of using contraception compared to a woman from Dhaka division (AOR: 0.83 and 0.42, 95% CI: 0.70-0.98 and 0.34-0.50 respectively). A woman from a rural setting had 23% less chance of contraception use than a woman from an urban setting (AOR: 0.77, 95% CI: 0.69-0.86). It was also found that a Muslim woman had 16% less chance of contraception use than a non-Muslim woman (AOR: 0.84, 95% CI: 0.73-0.98). A woman with 4 or more household members had approximately 2 times higher chance of contraception use than a woman with less than 4 household members (AOR: 1.83, 95% CI: 1.66-2.02).

Woman’s age, as expected, plays a vital role in contraception use. A 20-24 years aged women had 27% higher chance of using contraception than a 15-19 years aged women (AOR: 1.27, 95% CI: 1.10-1.47), whereas the chance was close to twice for a 25-29 years aged women (AOR: 1.87, 95% CI: 1.62-2.16) and more than 3 times higher for 30-34 and 35-39 years aged women (AOR: 3.37 and 3.23, 95% CI: 2.89-3.94 and 2.73-3.81 respectively). After age 39, the use of contraception decreased and just 78% higher for 40-44 years aged woman whereas it was 33% less for 45-49 years aged woman than a 15-19 years aged woman (AOR: 1.78 and 0.67, 95% CI: 1.50-2.10 and 0.56-0.80 respectively). Although women education had shown no significant association on contraception use, but their occupation had an emergent relation. An employed woman had 26% higher chance of using contraception than an unemployed woman (AOR: 1.26, 95% CI: 1.15-1.37).

It was found that an underweight or overweight woman had approximately 10% less chance of contraception use than a woman of normal weight whereas the chance was 29% less for an obese woman (AOR: 0.71, 95% CI: 0.60-0.84). The husband’s age difference with respondent and husband’s occupation had not shown significant association with contraception use, but surprisingly, husband’s education was found to be a significant determinant. A woman whose husband completed primary or secondary education had a more than 10% higher chance of contraception use than a woman whose husband had no schooling (AOR: 1.15 and 1.12, 95% CI: 1.04-1.28 and 1.01-1.25 respectively). If the husband completed more than secondary schooling then the chance of using contraception was increased and was approximately 30% higher than the husband with no schooling (AOR: 1.28, 95% CI: 1.12-1.46). As deemed, a woman whose husband was living with her as opposed to staying elsewhere had a nearly 3 times higher chance of using contraception (AOR: 2.78, 95% CI: 2.41-3.21), while if the spouse were sexually active in the past year, then there was a 5 times higher chance of contraception use than for moderate or no sexual activity (AOR: 4.97, 95% CI: 4.38-5.65). Although sex of household head was not significant, desire for children had a positive effect on use of contraception. When the husband desired fewer children then the chance of using contraception was 23% higher than both desired the same number (AOR: 1.23, 95% CI: 1.06-1.44).

A currently breastfeeding woman had more than twice the chance of contraception use compared to a woman currently not breastfeeding (AOR: 2.47, 95% CI: 2.12-2.87). Two other factors, amenorrheic status and abstaining status, significantly decreased the use of contraception. A woman who was amenorrheic had 16 times less chance of contraception use than a woman who was not amenorrheic (AOR: 0.06, 95% CI: 0.04-0.07), whereas a woman who was in the abstaining period had 6 times less chance of contraception use than a woman who was not in the abstaining period (AOR: 0.16, 95% CI: 0.10-0.23). Lastly, a woman who had only one child or 2+ children born in the last five years had more than twice the chance of contraception use compared to a woman who had no child born in the last five years (AOR: 2.44 and 2.12, 95% CI: 2.17-2.73 and 1.69-2.64 respectively). Furthermore, a woman who had only one child died had 14% less chance of contraception use than a woman who had no child died (AOR: 0.86, 95% CI: 0.76-0.97) whereas the chance is 28% less for that woman whose 2 or more children died (AOR: 0.72, 95% CI: 0.58-0.88).

## Discussion

**Fig. 5:**
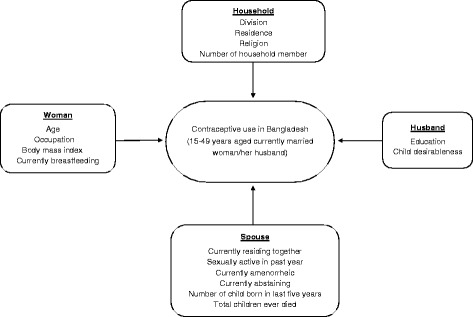
Interactive model to increase use of contraception in Bangladesh

Contraception is one of the key components for family planning while proper family planning can reduce mortality and morbidity of both child and mother [4, 11]. Our analysis provided a broader insight into the socio-economic, demographic, and other woman- and husband-related factors that influence the use of contraception in Bangladesh. We found that administrative division, place of residence, religion, number of household members, woman’s age, occupation, body mass index, breastfeeding practice, husband’s education, living status with wife, sexual activity in past year, woman’s desire for children, amenorrheic status, abstaining status, number of children born in the last five years and total children ever died were significantly influencing the use of contraception by women aged 15–49 years or their husbands in Bangladesh. An interactive model, as depicted in Fig. 5 and made in light of our findings, could therefore increase the contraceptive prevalence rate in Bangladesh if proper planning is implemented for the stakeholder.

The use of contraception varies geographically either with administrative or domain levels [19, 20, 38, 39]. We also found similar results where contraceptive use in Bangladesh varies with administrative divisions and place of residence. The rate of contraception use was higher in urban areas and inferior in Sylhet division. Many authors found that the selection of contraceptive methods depends on many psychological, social and cultural factors including perceptions of contraception and existing social norms about the culture [40, 41]. The use of contraception also varies with religious belief where belief about family planning and other cultural trends can decrease the prevalence of contraception use [40]. For example, abortion of a viable fetus is considered a serious crime equivalent to that of murder in Islam [42] while emergency contraception is also disapproved of [40], which causes less prevalence of contraception use by Muslim women. We found significant variation in contraception use according to religion: Muslim women were 16% less likely to use contraception than non-Muslim women. The use of contraceptive is highly related to family size [39, 43, 44]. Our study also found that the odds of using contraception was higher in those households which have 4 or more family members.

The current use of contraception varies by women’s age: the pill was the most widely used method among all women’s age groups except for those aged 45–49, who were more likely to use periodic abstinence. A similar picture was found for women aged 30–49 who were more likely to be sterilized than younger women. Islam [28] also found similar evidence that contraceptive prevalence rate was lower among younger women compared with older women from a cross-sectional study in Narsingdi district under Dhaka division. Many studies found that contraceptive use has the power to reduce fertility considerably and ultimately to improve maternal and child health. However, the use of contraception did not increase as level of women’s education increased, probably since males are the central decision maker of a family in Bangladesh. On the other hand, the odds of using contraception is positively associated with women’s occupation, where employed women are more likely to use contraception than unemployed women. Chuang et al. [45] and McNicholas et al. [46] had shown that sexually active obese women of reproductive age were significantly less likely to use contraception than women of normal weight. We also found that Bangladeshi obese women were 30% less likely to use contraception than women of normal weight. Although many studies had shown that husband’s age difference with wife is associated with contraceptive use [47, 48], we found that husband’s age difference with wife and husband’s education both play a vital role in contraceptive use, while husband occupation remains insignificant. As the husband’s level of education increases, the prevalence of contraceptive use increases since husband’s education level has influences the use of male sterilization and condoms [49], although men in any case have a significant role to play in the adoption of contraception if there is communication between a husband and wife on reproductive matters [50]. Hence, as might be expected, women currently residing with their husbands and sexually active in the past year had higher odds of using contraception.

Since males are predominantly the decision makers in Bangladesh, the husband’s desire for a child had a significant positive effect on contraception use, while for this reason media exposure concerning contraception use had not shown significant association. However, breastfeeding can be used as a birth control tool for many years, called the lactational amenorrhoea method, when after giving birth, a woman breastfeeds her baby exclusively. The act of breastfeeding naturally changes a woman’s hormones so that she does not become pregnant [51, 52]. We found that a woman who currently breastfeeds her child had more than twice the odds of using contraception. Meanwhile, the number of living children had not shown significant association with the use of contraception, since it is highly confounded with other factors such as woman’s age, number of children ever born and number of children born in the last five years. Interestingly, the effect of the variable ‘number of living children’ was completely nullified by the proxy variable, number of children born in the last five years. Similarly to Johnson and Sufian [53], we found that child mortality was associated with a lower rate of contraceptive use. Moreover, women who were amenorrheic and in the abstaining period had very little chance of using contraception, and hence we have adjusted these effects when identifying the factors that are truly associated with contraception use in Bangladesh.

This study has several strengths over other studies conducted in a similar setting. It followed a rigorous statistical technique by considering individual sampling weights when representing the actual nationwide result, and used mixed effect logistic regression modelling to identify correctly the factors associated with the use of contraception. Another strength of the study was that it considered a large number of potential factors that might have influenced contraception prevalence. There might be other broader factors related to contraceptive use, but to the best of our knowledge, our current model is the first rigorous model to identify a large number of covariates related to Bangladeshi women and their respective husbands that directly influence the use of contraception.

The main limitation of the study is that it is not possible to draw any temporal relationship between contraception use and its determinants since the data was cross-sectional in nature. A further analysis can be carried out by considering time variable so that factors that may affect the contraception use rate by time can be identified.

## Conclusion

The odds of women experiencing the outcome of interest are not independent due to the nested structure of the data, i.e. women from the same cluster typically share common exposure to community characteristics. As a result, a mixed effect model is implemented for the binary variable ‘contraceptive use’ to produce true estimates for the significant determinants of contraceptive use in Bangladesh. Knowing such true estimates is important for attaining future goals including increasing contraception use from 62 to 75% by 2020 by the Bangladesh government’s Health, Population & Nutrition Sector Development Program (HPNSDP). We anticipate that this framework shall help the policy makers and researchers to incorporate additional intervention or components in their existing family planning program since our model framework compiled all relevant factors that could potentially affect the use of contraception and would be useful to identify the gap of family planning related public health intervention.

## Additional file

Additional file 1 2014 Bangladesh Demographic and Health Survey data. Description of data: The Bangladesh Demographic Health Survey (BDHS) 2014 was a nationally representative cross sectional survey conducted between June and November 2014, covering the entire population of Bangladesh. The survey was based on a two-stage stratified sample of households. In the first stage, 600 enumerations areas (EAs) were selected with probability proportional to EA size, with 207 EAs from urban areas and 393 from rural areas, where an EA is either a village, a group of small villages, or a part of a large village. In the second stage, a systematic sample of 30 households on average was selected per EA to provide statistically reliable estimates of the key demographic and health variables for the country as a whole, for urban and rural areas separately and each of the seven divisions which are the administrative regions of Bangladesh. The 2014 BDHS used three types of questionnaires: a household questionnaire, a women questionnaire and a community questionnaire. For the women questionnaire, a total of 18,000 ever married and 16858 currently married (when conducting the interview) women aged between 15-49 years were interviewed. For our study, we considered only currently married women to ensure similar prevalence as BDHS 2014 reported. Further details about the data set can be found from Methods Section. (CSV 24,064 kb)

## Abbreviations

AOR: Adjusted odds ratio
BDHS: Bangladesh demographic and health survey
COR: Crude odds ratio
EA: Enumerations area
FP: Family planning
HPNSDP: Health, population & nutrition sector development program
